# SERRS Detection on Silver Nanoparticles Supported on Acid-Treated Melamine-Resin Microspheres

**DOI:** 10.3390/nano11051337

**Published:** 2021-05-19

**Authors:** Chaofeng Duan, Lu Shen, Yuqing Guo, Xiaogang Wang, Xiaohua Wang, Zhixian Hao

**Affiliations:** 1 Shanghai Key Laboratory of Chemical Assessment and Sustainability, School of Chemical Science and Engineering, Tongji University, Shanghai 200092, China; 1831027@tongji.edu.cn (C.D.); Shenlu218@163.com (L.S.); superyq@tongji.edu.cn (Y.G.); xgwang@tongji.edu.cn (X.W.); 2 Institute of Industrial Catalysis, Zhejiang University of Technology, Hangzhou 310014, China; wangxiaohua0909@163.com

**Keywords:** oligomers, melamine-resin microsphere, acetic acid treatment, SERRS substrate, pollutants detection

## Abstract

Melamine-resin microspheres were synthesized at a pH of 4.0 for 20 min and used as silver nanoparticle (AgNP) carriers for surface enhanced resonant Raman scattering (SERRS) detection. An acetic acid–treatment reaction was introduced into the fabrication of the final substrate. The SERRS performance of the substrate was effectively optimized by regulating excess formaldehyde and experimental parameters, such as acidity, number of treatments and reaction temperature in the acid-treatment reaction. Based on the SERRS detection, it was declared that a trace amount of oligomers with a certain degree of polymerization is necessary for the construction of SERRS hotspots. In addition, it is important to remove excess oligomers with reference to the synthetic reaction of the polymer materials, given the special role of oligomers and the wide application of polymer materials in SERRS detection.

## 1. Introduction

Nowadays, various polymer materials, such as polystyrene, polyamide, epoxy resin, phenolic resin, urea resin [1] and melamine resin [2,3], are frequently used in the fabrication of SERRS substrates. Therein, the inherent plasticity of these polymer materials was developed and utilized as much as possible. Their film or microsphere shape was used for adsorption of metal nanoparticles, their plane shape for metal colloidal lithography, their shell shape for encapsulation of nanoparticles [4,5,6,7,8], their molecularly imprinted membrane [9,10] for capturing specific analytes, their flexibility [11] for detecting contaminated fruits and their tensile state [12] or hollow spherical structure [13,14] for the effective construction of SERRS hotspots. In terms of molecular structures, the SERRS technology always takes advantages of some unique characteristics of polymer materials such as biocompatibility [15,16,17,18], multidentate coordination with metal nanoparticles [19,20], and three-dimensional construction of SERRS hotspots [19,21]. These characteristics depend on the abundance of selectable functional groups, such as –CN, –OH, –NH_2_ and –COOH, in their molecular structure. Although the self-assembly of metal nanoparticles is facilitated by multiple bonds between the metal and functional groups of the polymers [22,23,24,25], the polymer synthetic process may lead to contrasting functional group types. The confusion of these functional groups confers SERRS signals with serious reproducibility challenges that are intractable and generally concerning.

The urea and formaldehyde precipitation polymerization procedure [26] established that the output, crystallinity, morphology, density and water absorption capacity of the urea and formaldehyde resin (UF) microspheres were considerably dependent on the “excess monomer”, a general concept in copolymerization reactions. This concept emphasizes [26] the fact that a linear molecule A[BA]*_n_*BA, generated from the reaction with excess monomer A, for example, will be entirely different from the other molecule BA[BA]*_n_*B with the excess monomer B. Furthermore, the substrate incorporating either the UF resin [1] or melamine and formaldehyde resin (MF*_n_*, *n* = F/M molar ratio) microspheres [27] from excess formaldehyde are sensitive and stable in SERRS detection when compared to the SERRS-inert substrate that incorporates resin microspheres from excess urea or melamine. Wherein, MF*_n_* and UF microspheres are similar to each other in several aspects. Both reactions occur in acidic aqueous solutions, both products are resin microspheres, in both the NH_2_ groups at the terminals of polymer structures can be replaced with –NHCH_2_OH by the excess formaldehyde added in the reaction solution and, thus, both optimizations of the SERRS substrates have exactly the same characteristics [1,27]. In a recent study of DNA detection with the SERS substrate incorporating MF*_n_* microspheres [3], the parameter (*n*) was calculated and found to be 3.8, which is much larger than that of 1.5, a boundary between MF*_n_* microsphere characteristics based on its excess monomers [27]. This finding was in tandem with the effectiveness of SERRS substrate whose origin was the excess formaldehyde [1]. Herein, the biocompatibility and SERRS signal sensitivity of MF*_n_* microspheres was verified to be excellent.

In this study, MF*_n_* microspheres were pretreated with an acetic acid solution to support silver nanoparticles that was found to be much more effective in the R6G SERRS detection.

## 2. Experimental Methods

### 2.1. Synthetic and Acid-Treatment Reactions of Melamine-Resin Microspheres

MF*_n_* microspheres were synthesized through the precipitation polymerization of melamine and formaldehyde in acetic acid solution as previously described [28]. Briefly, a mixture of 90.0 mL acetic acid solution at a pH of 4.0, 2.50 g of melamine and 1.60 mL of 38 wt % formaldehyde was prepared in a conical flask and sealed with a piece of plastic membrane. The conical flask was placed in a water bath and dissolved at 65 °C, under electromagnetic stirring. It was then kept at 65 °C for 20 min until a cloudy state appeared. The mixture was then cooled and separated through a Buchner funnel, along with a washing step using water and ethanol, respectively. After drying at room temperatures in a desiccator for more than 24 h, the obtained MF*_n_* microspheres were subsequently used for SERRS-substrate fabrication. In the synthetic reaction, the F/M molar ratio was expressed as a parameter (*n*) and equaled 1.00. From this ratio, other values were determined.

Except for replacement of the reactants by MF*_n_* microspheres, as well as an ultrasonic dispersion step at the initial stage, the acid-treatment reaction of MF*_n_* microspheres was performed under the same conditions as in their synthetic reaction. In this study, any experimental parameters, including temperature, time, number of acid-treatments and acidity, as well as the volume of the acetic acid solution in the acid-treatment reaction, could be designed as a variable to examine the performance of the SERRS substrate incorporating the MF*_n_* microspheres.

### 2.2. Synthesis of Silver Nanoparticle Colloid Solution

The silver nanoparticle (AgNP) colloid solution was synthesized through the reduction of silver nitrate by sodium citrate [1,29]. Then 95 mL of silver nitrate solution in a glass beaker was heated to boiling and 5.0 mL of sodium citrate added. The glass beaker was then sealed immediately using a piece of plastic membrane and left to boil for an additional 30 min. A stable gray-green AgNP colloid solution was formed and left to cool down to room temperature. The optimized molar ratio between silver nitrate and sodium citrate was 2:3, and their initial concentrations in the silver colloid solution were 2.0 × 10^−3^ and 3.0 × 10^−3^ M, respectively. The water used in AgNP colloid solution synthesis and SERRS-substrate fabrication had an electrical conductivity of 1.37 µS/cm. All chemicals were of analytical grade and were obtained from Sinopharm Chemical Reagent Co., Ltd., Shanghai, China.

### 2.3. Fabrication and Incubation of SERRS Substrate

Prior to R6G incubation, SERRS-substrate fabrication was performed in situ in a 2 mL centrifuge tube. AgNPs were separated from 2.0 mL of colloid solution through two sequential centrifugation steps at 2000 and 6000 rpms for 5 min. They were re-dispersed in water to the initial volume. The precipitate from the first step and the supernatant from the second step were discarded. Finally, 5.0 mg of MF*_n_* microspheres were impregnated in the AgNP solution for 60 min and separated at 2000 rpm to complete the SERRS-substrate fabrication. The AgNPs adsorbed on MF*_n_* (AgNP/MF*_n_*) were invariably separated and used as a whole for SERRS detection.

During R6G incubation, the SERRS substrate was dispersed and impregnated in a 1.00 × 10^−7^ M R6G solution for 2.0 h. After separation at 2000 rpm, the incubated substrate was transferred onto a glass slide that had been pretreated with ethanol (three times) and dried in a desiccator for 12 h. Moreover, other analytes, such as tetramethylthiuram disulfide, malachite green, p-hydroxythiophenol, basic violet 14, etc., in 1.00 × 10^−5^ M aqueous solutions, were examined as described above on an optimized substrate to verify the SERRS performance.

### 2.4. Characterization and SERRS Detection

SERRS signals were collected on a confocal microscope Raman spectrometer (Invia Reflex Renishaw, Renishaw Apply Innovation, Gloucestershire, UK) with a 514.5 nm and 0.2 mW laser, an acquisition time of 10 s and a wave-number region from 4000 to 200 cm^−1^. The sample was treated through various steps, such as being dispersed in ethanol, dropped onto a silicon wafer, dried at room temperature and being sprayed with powdered gold. The morphology of the sample was determined using a scanning electron microscope (SEM, EFI Quanta 250 FEG USA), where the accelerating voltage was set at 20.0 kV.

## 3. Results and Discussion

### 3.1. Synthetic and Acid-Treatment Reactions of Melamine-Resin Microspheres

The AgNP colloid solution and MF*_n_* microspheres were synthesized separately while the SERRS substrate, i.e., AgNP/MF*_n_*, was fabricated through the adsorption of AgNPs on the MF*_n_* in a 2 mL centrifuge tube. Incubation with R6G was performed in the same tube. The SERRS performance of AgNP/MF*_n_* was shown to depend on parameter (n), i.e., the F/M molar ratio adopted in the MF*_n_* synthetic reaction. SERRS enhancement on AgNP/MF*_n_*_>1.5_ was attributed to the abundance of –NHCH_2_OH groups on the MF*_n_* surface that originated from excess formaldehyde [27] that had been added in the MF*_n_* synthetic reaction. It was also found that as the MF*_n_* synthetic-reaction time increased, the SERRS substrate was activated in a progressive way, even though the parameter (n) was higher than 1.5, a boundary value of the MF*_n_* characteristics. In a separate experiment, R6G-SERRS enhancement was observed on a set of pure AgNPs at a laser power of 0.01 mW only if they were treated with the MF*_n_*-impregnation liquor. The liquor was the supernatant that had been centrifuged from impregnation mixture of the MF*_n_*_>1.5_ in pure water. Therefore, a certain amount of polymers with low polymerization degrees (oligomers) must dissolve in water and adsorbed on the AgNPs to exhibit their role in SERRS detection.

**Figure 1:**
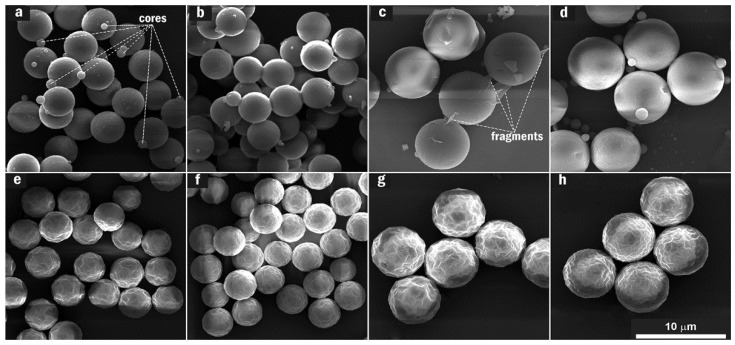
Impact of the acid-treatment reaction on MF*_n_* microsphere SEM images: (**a**) MF_2_, (**b**) MF_3_, (**c**) MF_6_ and (**d**) MF_9_ at the top were collected on non-treated microspheres from a synthetic reaction at a pH of 4.0 and 65 °C for 20 min only; meanwhile (**e**) MF_2_, (**f**) MF_3_, (**g**) MF_6_ and (**h**) MF_9_ at the bottom were collected on the microspheres treated in an acetic acid solution at a pH of 4.0 and 60 °C for 20 min. The MF*_n_* microsphere surfaces had been transformed into shrinkage textures while the resin cores and fragments had all been cleaned up in the acid-treatment reaction.

Since it was able to catalyze the MF*_n_* synthetic reaction (a precipitation polymerization reaction) that involved the nucleation, growth and maturation of the MF*_n_* microspheres, the acetic acid solution was expected to do something more in the fabrication of the SERRS substrate. Therefore, a set of MF*_n_* acid-treatment reactions were designed with reference to the MF*_n_* synthetic reaction. A series of SEM micrographs of MF*_n_* microspheres with *n* = 2.0–9.0, having been synthesized at a pH of 4.0 and at a temperature of 65 °C for 20 min, were displayed at the top (a–d), while the series at the bottom (e–h) was of the samples that had been treated in the acetic acid solution at a pH of 4.0 and temperatures of 60 °C for 20 min, as shown in Figure 1. A short synthetic-reaction time was unfavorable for fabrication of the SERRS substrate and was herein adopted so as to highlight advantages of the acid treatment method. Except the temperatures, which decreased by 5 °C, the experimental parameters in the acid-treatment reaction were consistent with those adopted in the MF*_n_* synthetic reaction process. Besides some resin cores with ~1.0 μm sizes and some fragments in the non-treated microspheres, two single microsphere sizes (~3.80 and ~6.10 μm) corresponded to the parameters *n* ≤ 3.0 and ≥6.0, respectively (Figure 1a–d). Resin cores were generated in the primary stage of the MF*_n_* synthetic reaction and disappeared as the reaction time increased. Resin fragments (originating from partial dissolution of the MF*_n_* microspheres in the ethanol solvent) formed in ethanol volatilization during sample preparation for SEM tests or separation from their synthetic-reaction mixture.

As shown in Figure 1e–h, the acid-treatment reaction revealed some essential characteristics of the oligomers in MF*_n_* microspheres. The surface of the MF*_n_* microspheres exhibited a shrinkage texture that was attributed to the physical-shrinkage phenomenon of the microspheres during desiccation, after a certain amount of oligomers had been removed in the acid-treatment reaction. Furthermore, the resin cores and fragments were all cleaned up. There were obviously two other forms of the oligomers in MF*_n_* microspheres that were much more produced at the primary stage of the MF*_n_* synthetic reaction and dissolved or/and hydrolyzed into the acetic acid solution.

Table 1 shows MF*_n_* microsphere mass loss rates in the acid-treatment reaction. The mass loss rate for all samples was more than 4.29 wt %, and varied with parameter (*n*). In the case of M_2_F, the loss rate reached a maximum 17.5 wt %, wherein the MF*_n_* synthetic reaction was executed at *n* = 0.5 and the melamine in the synthetic reaction process was too much to produce a SERRS-active substrate.

Supplementary Materials Figure S1 provides the Raman spectra of MF*_n_* microspheres and the microspheres after the acid-treatment reaction. There existed a progressive addition of formaldehyde into the MF*_n_* microspheres if the parameter (*n*) increased and, meanwhile, the removed mass portion more than 4.29 wt %, as shown in Table 1, could be contributed to MF*_n_* oligomers with more terminal –NHCH_2_OH groups. The impact of the polymerization degree on the F/M molar ratio (*n*) in MF*_n_* structure is shown Supplementary Materials Figures S2 and S6, while the terminal overreaction of excess formaldehyde is presented in the supplementary information, as shown in Equations LO, PO and TO, as well as Supplementary Materials Figures S7–S12.

### 3.2. SERRS-Substrate Fabrication/Adsorbing Silver Nanoparticles on Melamine-Resin Microspheres

The SERRS-substrate fabrication and incubation with R6G were sequentially executed in a 2 mL centrifugation tube in situ. The silver colloid solution was prepared by boiling a solution mixed with silver nitrate and sodium citrate. The solution was then purified by centrifugation at a speed range of 1000 to 6000 rpm before use. The MF*_n_* microspheres were added into the silver colloid solution to bind AgNPs on their surfaces and finally form the hybrid microspheres.

**Figure 2:**
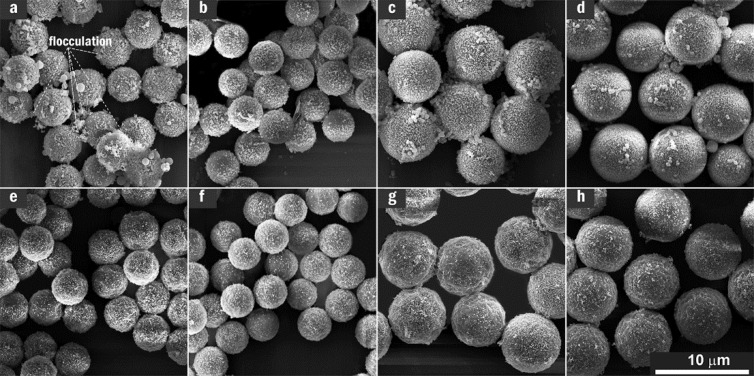
Impact of the acetic acid treatment on SEM images of AgNP/MF*_n_* substrate: (**a**) AgNP/MF_2_, (**b**) AgNP/MF_3_, (**c**) AgNP/MF_6_ and (**d**) AgNP/MF_9_ at the top were collected on the substrate incorporating non-treated microspheres; (**e**) AgNP/MF_2_, (**f**) AgNP/MF_3_, (**g**) AgNP/MF_6_ and (**h**) AgNP/MF_9_ at the bottom collected on the substrate incorporating the acid-treated microspheres, wherein the AgNPs looked aggregated in a flocculation way on the non-treated MF*_n_* microspheres.

SEM micrographs of a set of AgNP/MF*_n_* microspheres incorporating non-treated and the acid-treated MF*_n_* microspheres are respectively presented at the top (a,b,c,d) and bottom (e,f,g,h) of Figure 2. It was found that AgNPs were heavily adsorbed and aggregated on the non-treated MF*_n_* microspheres involving the resin cores as shown at the top row of Figure 2. However, they were delicately dispersed on the acid-treated microspheres as shown at the bottom of Figure 2.

**Figure 3:**
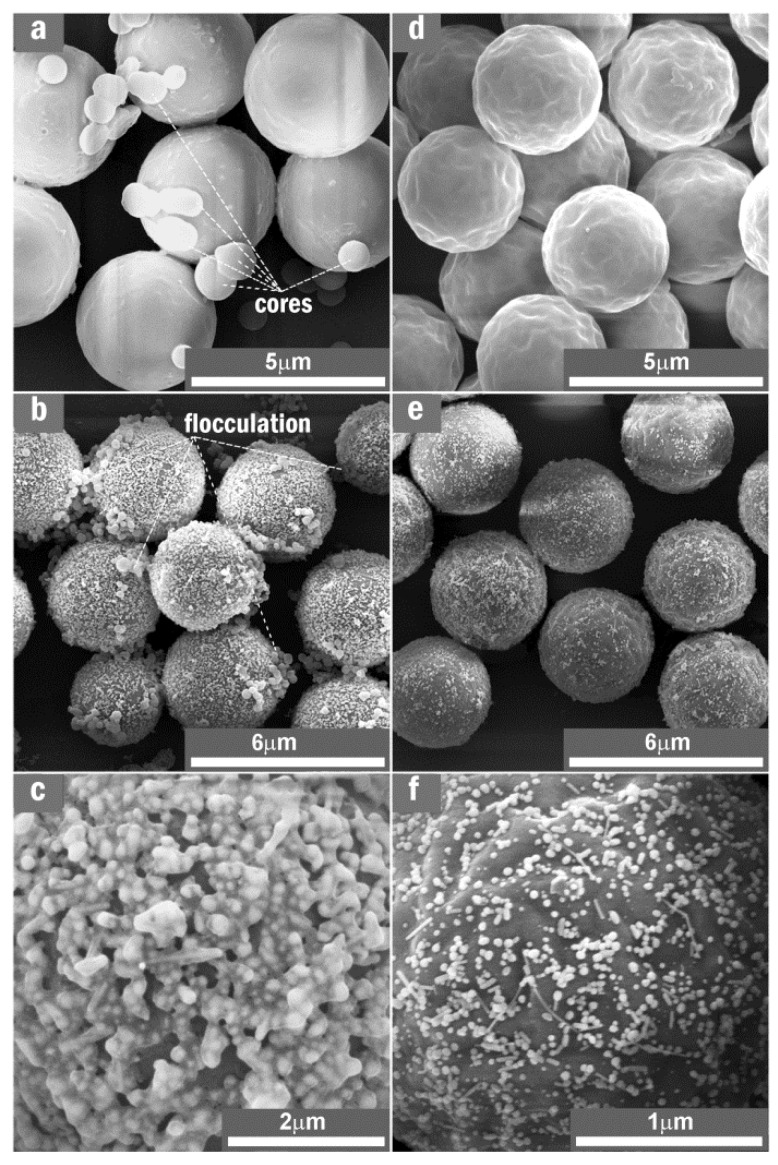
Impact of the acid-treatment reaction on SEM images of the MF_3_ microspheres and AgNP/MF_3_ substrate: (**a**) and (**d**) were collected on non-treated and the acid-treated MF_3_ microspheres; (**b**) and (**e**) were collected on substrates incorporating the MF_3_ samples of (**a**) and (**d**); and (**c**) and (**f**) were amplified from (**b**) and (**e**), respectively. After the acid-treatment reaction, the AgNPs were well dispersed on the surface of the microsphere.

The impact of MF_3_ acid-treatment reaction on morphologies of MF_3_ microsphere and AgNP/MF_3_ substrate could be summarized in Figure 3. The oligomer quantity in the MF_3_ microsphere (Figure 3a) was 6.21 wt% more than that in the acid-treated ones (Figure 3d). AgNPs on the microspheres (Figure 3b) were bound by the oligomers in a flocculation way when compared to those on the acid-treated microspheres (Figure 3e). The oligomers exhibited an excessive overflow and an intensive interaction with AgNPs through the simple SERRS-substrate fabrication under aqueous solution. The AgNPs were restricted in the colloid capsules that had been made from the oligomers as shown in Figure 3c. The sizes of AgNP capsules on the non-treated MF_3_ microsphere (Figure 3c) were as high as ~300 nm, which was ~5 times that of the AgNPs on the acid-treated one (Figure 3f). The effectiveness of acid-treatment reaction in MF_3_ oligomer desorption and solubility in the acetic acid solution was shown by the excellent dispersion of the AgNPs on the acid-treated microsphere as shown in Figure 3f. Herein the silver content in the substrate incorporating no-treated MF_3_ microspheres was 91 ppm and this value in the substrate incorporating acid-treated ones was only 58 ppm, which were detected on spectrophotometry at a 400 nm wavelength and summarized in Supplementary Materials Figure S13 and Table S1. It is obvious that a larger amount of AgNPs adsorbed on the no-treated MF_3_ microspheres was due to their more amount of oligomers.

### 3.3. Performance of the SERRS Substrate Incorporating the Acid-Treated Melamine-Resin Microspheres

**Figure 4:**
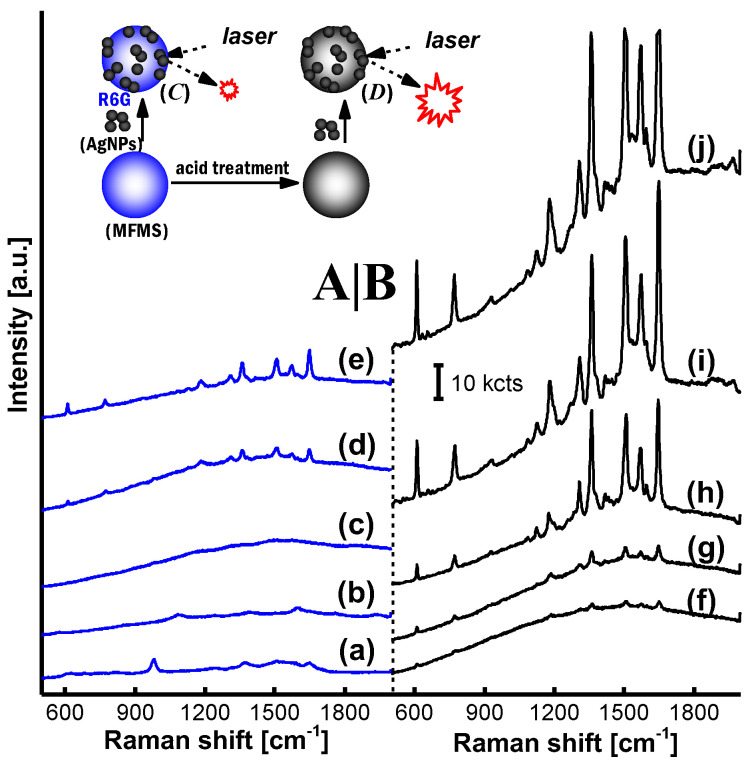
Impact of the acid-treatment reaction on R6G-SERRS spectra on the AgNP/MF*_n_* substrate: (**a**) AgNP/MF_1_, (**b**) AgNP/MF_2_, (**c**) AgNP/MF_3_, (**d**) AgNP/MF_6_ and (**e**) AgNP/MF_9_ in (**A**) were collected on the substrate incorporating non-treated microspheres at a laser power of 0.2 mW; meanwhile (**f**) AgNP/MF_1_, (**g**) AgNP/MF_2_, (**h**) AgNP/MF_3_, (**i**) AgNP/MF_6_ and (**j**) AgNP/MF_9_ in (**B**) were collected on the substrate incorporating the acid-treated microspheres at a laser power of 0.01 mW. The blank AgNPs was examined at a laser power of 0.05 mW and the R6G-SERRS signal was magnified 3 times as shown at the bottom of (**B**). As shown in the inset, SERRS signals on the substrate had their intensities enhanced if the incorporated MF*_n_* microspheres were pretreated in acetic acid solution. Furthermore, superiority of excess formaldehyde in SERRS detection was noticeable as the parameter (n) increased.

R6G-SERRS spectra on the AgNP/MF*_n_*_=1–9_ incorporating the non-treated and the acid-treated MF*_n_* microspheres are shown in Figure 4A,B, respectively. The efficiency of R6G-SERRS was inhibited on the AgNP/MF*_n_* substrate incorporating the non-treated MF*_n_*, as shown in Figure 4A. The advantages of excess formaldehyde were only exhibited by some faint SERRS signals when the parameter (n) was equal to 6.00 or 9.00, much more than the boundary (1.50) of MF*_n_* characteristics. However, R6G-SERRS signals exhibited a heightened intensity on the substrate incorporating the acid-treated MF*_n_* microspheres when the parameter (*n*) increased from 1.00 to 9.00, as shown from Figure 4B(f–j), where the acid-treatment reaction was performed at a pH of 4.0 and at a temperature of 60 °C for 20 min.

The concept “excess formaldehyde” that is favorable to the R6G-SERRS performance was encountered again on the AgNP/MF*_n_* with the parameter *n* = 3.0–9.0, although the MF*_n_* synthetic reaction was only kept for 20 min this time, much shorter than 9.0 h adopted in our previous study in absence of the MF*_n_* acid-treatment reaction. The MF*_n_* characteristic boundary based on the parameter (n) is shown in the supplementary information. The 0.01 mW laser power was utilized for SERRS examination of the substrate incorporating the acid-treated MF*_n_* microspheres. It was 5% of the power adopted on the substrate incorporating the non-treated MF*_n_* microspheres as shown in Figure 4A. The oligomers at more than 4.29wt % prevented the AgNPs from constructing the SERRS hotspots on the non-treated MF_*n*=3–9_ microspheres. Herein, the blank AgNPs was examined at a laser power of 0.05 mW that was 5 times of the 0.01 mW used on the substrate incorporating acid-treated microspheres. Even though it was magnified 3 times, the R6G-SERRS signal was still faint and not comparable to that obtained on the acid-treated AgNP/MF_*n*=3–9_ microspheres. It is obvious that the R6G concentration of 10^−7^ M was just a detection limit based on the blank AgNPs and this SERRS method.

**Figure 5:**
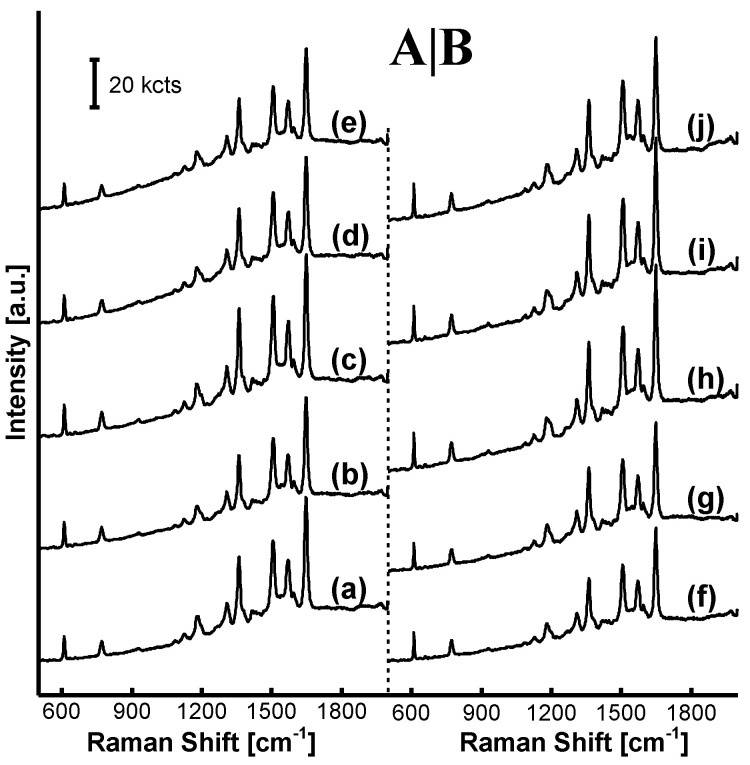
SERRS spectra of AgNP/MF_6_ vs. treatment time and acid-volume in the MF_6_ acid-treatment reaction: (**a**) 10, (**b**) 20, (**c**) 40, (**d**) 60 and (**e**) 80 min in (**A**) were collected on the substrate incorporating the acid-treated MF_6_ microspheres at varied treatment times, from 10 to 80 min; meanwhile, (**f**) 1×, (**g**) 2×, (**h**) 3×, (**i**) 4× and (**j**) 5× times in (**B**) were collected on the substrate incorporating the acid-treated MF_6_ microspheres at varied acetic acid volumes, from 1× to 5× times 90 mL. The SERRS performance of the substrate was nearly stable.

The impact of reaction time and the acetic acid volume in the MF_6_ acid-treatment reaction on AgNP/MF_6_ SERRS performance is shown in Figure 5A,B respectively, where the parameter (*n*) was chosen as a constant 6.0 in the MF*_n_* synthetic reaction. The R6G-SERRS signals from all samples exhibited a remarkable intensity and neither the reaction time nor the acetic acid volume could impact SERRS performance of the AgNP/MF_6_ in the MF_6_ acid-treatment reaction (Figure 5). The acid-treatment time was extended to 80 min while the acetic acid volume was increased to 450 mL, equaling 4 and 5 times those adopted in the MF_6_ synthetic reaction, respectively.

A possible explanation for these results was that there is a dependency between the polymerization degree of oligomers and the acidity of the acetic acid solution. The oligomers in the MF_6_ microspheres with a polymerization degree that was no more than a certain value would, therefore, be removed under a determinate acidity.

**Figure 6:**
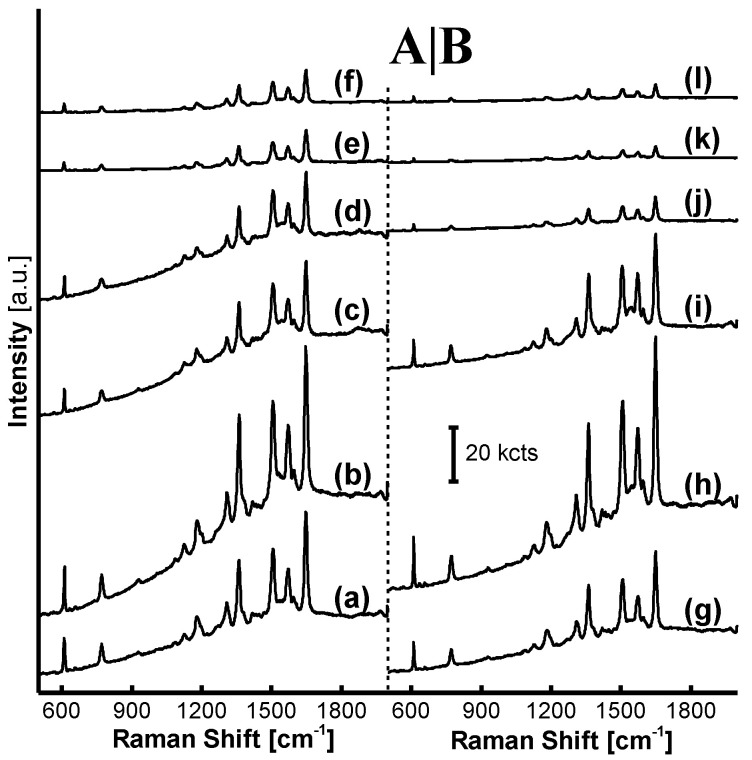
SERRS spectra of AgNP/MF_6_ vs. the acidity and treatment number in the MF_6_ acid-treatment reaction: (**a**) pH 4.0, (**b**) 3.7, (**c**) 3.3, (**d**) 3.0, (**e**) 2.7 and (**f**) 2.3 in (**A**) were collected on the substrate incorporating MF_6_ microspheres that had been treated at varied acidic pH values, from 4.0 to 2.3; meanwhile (**g**) 1, (**h**) 2, (**i**) 3, (**j**) 4, (**k**) 5 and (**l**) 6 times in (**B**) were collected on the substrate incorporating the microspheres that had been treated with a variable treatment number from 1 to 5 times. The SERRS performance of the substrate could be optimized by variation of the acidity or treatment number in the MF*_n_* acid-treatment reaction.

The impact of acetic acid and the number of treatments in the MF*_n_* acid-treatment reaction on AgNP/MF_6_ SERRS performance is shown in Figure 6A,B, respectively. As the acidity increased from a pH of 4.0 to 3.7, AgNP/MF_6_ SERRS signals were enhanced; however, they were rapidly weakened as the acidity increased from a pH of 3.7 to 2.3, as shown in Figure 6A(a–f). In addition, the AgNP/MF_6_ SERRS signal intensity was at a maximum when the number of acid treatments increased from one to two. However, the intensity declined rapidly as the treatment number increased from two to six, as shown from Figure 6B(g–l).

If there is a dependency between the oligomer polymerization-degree and acidity in the MF_6_ acid-treatment reaction, it is attributed to the fact that both acidity and the number of acid-treatments in the MF*_n_* acid-treatment reaction can be used to optimize SERRS performance. Therefore, a trace amount of oligomers with a determinate polymerization degree is necessary for R6G-SERRS detection.

**Figure 7:**
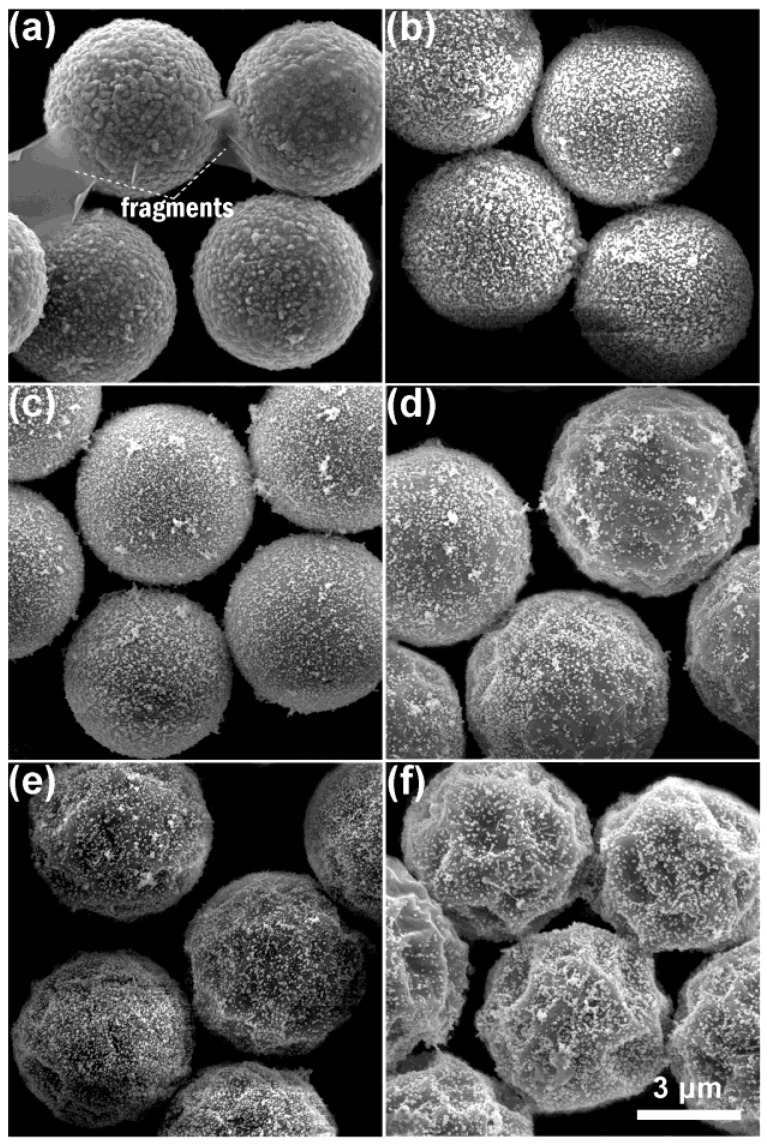
Impact of the MF_6_ acid-treatment temperature on SEM images of the AgNP/MF_6_ substrate: (**a**) 0, (**b**) 20, (**c**) 40, (**d**) 60, (**e**) 80 and (**f**) 100 °C were collected on the substrate incorporating the MF_6_ microspheres that had been previously treated at varied acid-treatment temperatures, from 0 to 100 °C. The characteristic shrinkage textures on MF_6_ microsphere surfaces became gradually prominent as the acid-treatment temperature increased from 60 to 100 °C.

The impact of the acid-treatment temperature on the morphology of the AgNP/MF_6_ is shown in Figure 7, where the MF_6_ acid-treatment temperature increased from 0 to 100 °C. With the acid-treatment temperature increasing from 0 to 100 °C, the characteristic shrinkage textures on MF_6_ microsphere surfaces became more prominent while the AgNPs were progressively dispersed. Resin fragments on the AgNP/MF_6_ appeared when the MF_6_ acid-treatment reaction was performed at 0 °C as shown in Figure 7a. This implies that the MF*_n_* acid-treatment reaction was activated at a high temperature. It was clear that the characteristic shrinkage textural morphology was initiated at the acid-treatment temperature of 60 °C as shown in Figure 7d. This characteristic temperature is significant for the effective removal of oligomers in the MF_6_ acid-treatment reaction. This temperature is close to the MF*_n_* synthetic-reaction temperature of 65 °C, and it, therefore, verifies that the generation and desorption of the MF*_n_* oligomers are reversible in the precipitation polymerization of MF*_n_* microspheres. The mass loss rates of MF_6_ in the acid-treatment reaction at the different acid-treatment temperatures were shown in Table 2. With the temperature increasing the mass loss rates of MF_6_ increased from 3.90% to 5.10%.

**Figure 8:**
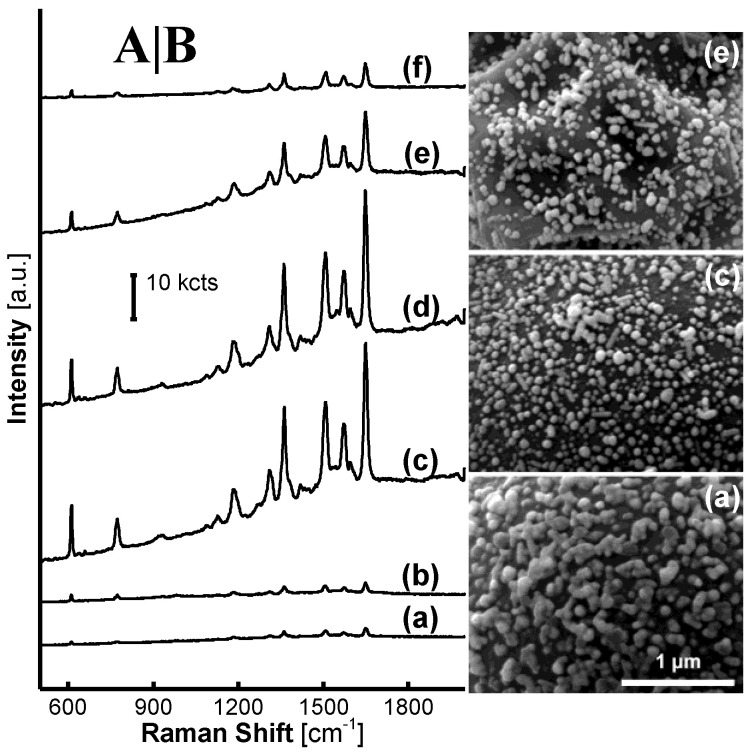
Impact of the MF_6_ acid-treatment temperature on R6G-SERRS spectra on AgNP/MF_6_ substrate: (**a**) 0, (**b**) 20, (**c**) 40, (**d**) 60, (**e**) 80 and (**f**) 100 °C in (**A**) were collected on the substrate incorporating the MF_6_ microspheres previously treated at varied acid-treatment temperatures, from 0 to 100 °C. The SEM images of the samples in (**a**,**c**,**e**) are presented in (**B**). The AgNPs were encapsulated in the oligomer shells as the acid-treatment reaction was executed at low temperatures. (**a**,**c**,**e**) in Raman spectroscopy and (**a**,**c**,**e**) in SEM are two characterization methods of the same substance.

Figure 8 shows the impact of MF_6_ acid-treatment temperature on the SERRS performance of AgNP/MF_6_. The activity of AgNP/MF_6_ was inhibited in R6G-SERRS detection when the MF_6_ acid-treatment reaction was performed at 0 and 20 °C as shown in Figure 8A(a,b). In addition, SERRS performance rapidly declined as the MF*_n_* acid-treatment temperature increased from 60 to 100 °C, as shown in Figure 8A(d–f). These results emphasize an important fact that the more and insufficient oligomers on the surface of MF*_n_* microsphere were both unfavorable to the SERRS fabrication.

Supplementary Materials Figure S14 shows the R6G-SERRS performance of AgNPs treated with the liquor from the MF_6_ acid-treatment reaction. The R6G SERRS signal was enhanced with the acid-treatment temperature gradually increased from 0 to 100 °C. These results were on the contrary with those obtained on the AgNP/MF_6_ where the incorporating MF_6_ from the same acid-treatment reactions executed at the varied temperature from 40 to 100 °C. It was obvious that the oligomers in the MF*_n_* microspheres had transferred into the hydrolyzed liquor and played an important role in the construction of SERRS hotspots.

The MF*_n_* microspheres were synthesized with melamine and formaldehyde under catalysis of an acetic acid solution, in which the MF*_n_* oligomers were formed, grew, aggregated and precipitated into the MF*_n_* microspheres. Herein, the acetic acid-treatment reaction of MF*_n_* microspheres could be considered as a reversible procedure of this aggregation reaction in absence of the reactants melamine and formaldehyde. It is obvious that the degree of presence or absence of the oligomers on the surface of an MF*_n_* microsphere or in an acidic hydrolyzed liquor resulted in an opposite trend in the SERRS response of the AgNPs, as seen when comparing Figure 8A with Supplementary Materials Figure S14. These oligomers could be a linear, planar, or three-dimensional structure as illustrated in Supplementary Materials or in our previous works [1,26,27]. The chemical group –NHCH_2_OH at the ends of an oligomer structure was favorable to the formation of SERRS hotspots [1,27], which was as same as summarized on UF microspheres [1].

The MF*_n_* synthetic-reaction temperature was valuable to the MF*_n_* acid-treatment reaction. The remarkable SERRS enhancement on the AgNP/MF_6_ that incorporated the acid-treated MF_6_ microspheres was exhibited at 40–60 °C, as shown in Figure 8A(c,d), slightly less than the MF_6_ synthetic-reaction temperature of 65 °C. We hypothesize that oligomers should work within an appropriate amount and a certain polymerization degree. Herein, the AgNP nano-capsules and the shrinkage textural morphology represents two extreme conditions, where the oligomers were much more or poor on the surface of the microspheres that were unfavorable to the construction of SERRS hotspots, as shown in Figure 8B(a,e), respectively.

**Figure 9:**
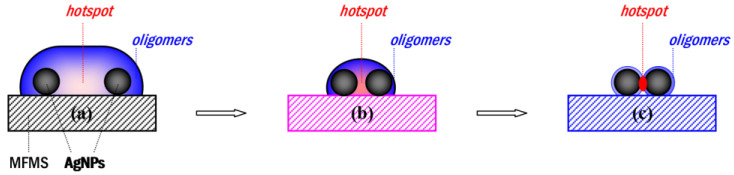
A schematic on the formation of the SERRS hotspots with MF oligomers participation: (**a**) a gap between two AgNPs is swollen by oligomers, (**b**) becomes small with the amount of oligomers decreased, and (**c**) works as an activated SERRS hotspot with the oligomers further decreased after the acid-treatment reaction.

Although a rough surface of the MF_6_ microspheres could be produced at some harsh acid-treatment parameters, such as the treatment temperature 100 °C or pH 2.3, the SERRS substrate could not provide a higher SERRS activity. Therefore, the oligomers must play an important role in the formation of the SERRS hotspots. Based on the above results, a schematic on the formation of the SERRS hotspots is provided in Figure 9.

A large oligomer capsule contains a pair of AgNPs (a) was formed on a MF microsphere, which becomes small, as the amount of the oligomers decreases in the acid-treatment reaction (b), and an active SERRS hotspot has finally formed at the gap between the AgNPs, with a further decrease of oligomers within the acid-treatment reaction at 60 °C (c).

The gap between two AgNPs is swollen with a large amount of oligomers on a MF_6_ resin microsphere, as shown in Figure 9a, and becomes narrow with the amount of the oligomers decreased following the acetic acid–treatment reaction, as shown in Figure 9b. It is finally activated and acts as a SERRS hotspot [30,31] in the R6G detection when its oligomers contained in is further decreased within the acid-treatment reaction at 60 °C for 20 min, as shown in Figure 9c. Therein, oligomers are necessary for the formation of SERRS hotspots and work as a linker [30,31] to hold the AgNPs to close to each other.

**Figure 10:**
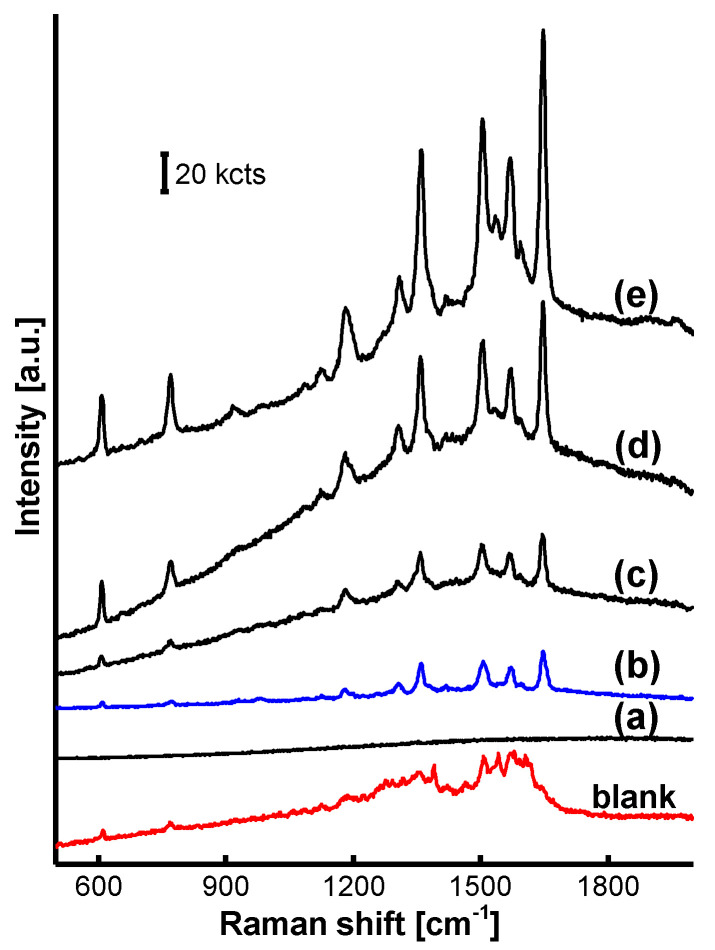
SERRS spectrum as a function of the R6G concentration on the optimized AgNP/MF_6_: (**a**) 1.0 × 10^−15^, (**b**) 1.0 × 10^−13^, (**c**) 1.0 × 10^−11^, (**d**) 1.0 × 10^−9^ and (**e**) 1.0 × 10^−7^ M were R6G-SERRS spectra on the optimized AgNP/MF_6_ treated with a R6G solution from 1.0 × 10^−15^ to 1.0 × 10^−7^ M R6G at a laser power of 0.01 mW and the SERRS detection was conducted on the blank AgNPs treated with 1.0 × 10^−7^ M R6G solution at a laser power of 0.05 mW. The R6G SERRS spectrum was still clear if the AgNP/MF_6_ was treated with a 1.0 × 10^−13^ M R6G solution.

The SERRS spectrum on AgNP/MF_6_, incorporating the acid-treated MF_6_ microspheres, varied with the R6G concentration from 1.0 × 10^−15^ to 1.0 × 10^−7^ M was provided in Figure 10. Its detection limit was lower than 1.0 × 10^−13^ M, where the R6G characteristic peaks were still clear as shown in Figure 10b. The SERRS detection on the blank AgNPs treated with 1.0 × 10^−7^ M R6G was conducted at a laser power of 0.05 mW that was five times the value 0.01 mW adopted on AgNP/MF_6_. Obviously, the blank used for 10^−7^ M R6G was incomparable to the optimized AgNP/MF_6_ for 10^−13^ M R6G, even though it was conducted at a much high laser power of 0.05 mW. The R6G detection limit on the optimized AgNP/MF_6_ was thus decreased to less than 10^−6^ times it on the blank according to these quantitative results.

**Figure 11:**
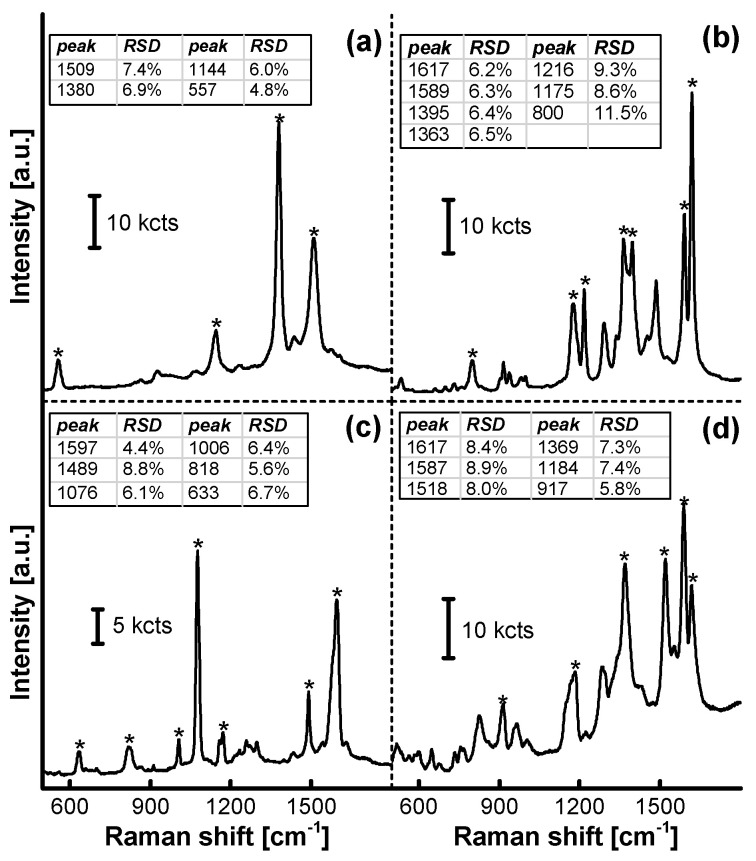
SERRS spectra from a set of analytes on AgNPs/MF_6_. (**a**) Tetramethylthiuram disulfide (LDL: 1.0 × 10^−8^ M. CAS: 137-26-8; disinfectant, mildew preventive, pesticide, residue over seven days and vulcanization accelerator in rubbers); (**b**) malachite green (LDL: 1.0 × 10^−7^ M. CAS: 2437-29-8; dye and toxic antiseptic for aquaculture); (**c**) p-hydroxythiophenol (LDL: 1.0 × 10^−7^ M. CAS: 637-89-8; pharmaceutical and dye intermediate); (**d**) basic violet 14 (LDL: 1.0 × 10^−9^ M. CAS: 632-99-5; dye). Each concentration of the analytes was 1.00 × 10^−5^ M, the MF_6_ in SERRS substrate was synthesized at *n* = 6.00, pH = 4.0, and at a temperature of 65 °C for 20 min and acid-treated at a pH of 4.00 and at a temperature of 40 °C for 10 min. The position of the Raman peak of each material is marked with “*”. The detection limits for each analyte are shown in Supplementary Materials Figures S16–S19.

SERRS spectra of a set of organic analytes on AgNPs/MF_6_ are shown in Figure 11 and Supplementary Materials Figure S15. The characteristic peaks in each SERRS spectrum were consistent with those in the standard spectrum. The relative standard deviations of the peak strengths from twelve tests on the SERRS substrate (see Supplementary Materials Figure S15) were not more than 12%, as shown in each set of spectrum in Figure 11. The AgNP/MF*_n_* substrate has, therefore, been incorporated into the SERRS analyses of some environmental pollutants, after the incorporated MF*_n_* microspheres were treated in the acetic acid solution.

## 4. Conclusions

Melamine-resin microspheres that were synthesized for 20 min were proved to be available in the fabrication of SERRS substrate, only if they were treated with an acetic acid solution before supporting silver nanoparticles. The performance of the SERRS substrate, incorporating in the melamine-resin microspheres with excess formaldehyde, can be optimized by regulating experimental parameters of the acetic acid–treatment reaction. Herein, the best *n* = F/M is 6, the best pH = 3.7, the best temperatures of acid treatment is 60 °C and the best condition for forming the hotspots for the loading temperature of silver nanoparticles is 40 °C. The R6G SERRS detection limit was equal to 10^−13^ M that was 10^6^ times the value detected on the blank AgNPs. These advantages in SERRS substrate incorporating the acid-treated melamine-resin microspheres are attributed to the role of the trace amounts of oligomers with certain polymerization degrees. Although the 4.29–6.21 wt % of their content in the melamine-resin microspheres was found to be excessive, the oligomers in trace amounts are found to be indispensable for the SERRS detection. Oligomers are found in many polymers, and what a role they will play and how to deal with them may be the crucial issues for the SERRS fabrication. Therefore, effectiveness of the acid treatment of the oligomers in melamine-resin microspheres has just shown their significance in the SERRS detection.

## Figures and Tables

**Table 1:** Mass loss rates of MF*_n_* microspheres in the acid-treatment reaction.

Samples	M_2_F	MF_3_	MF_6_	MF_9_
**parameter *n***	0.500	3.00	6.00	9.00
* mass loss rate (wt %)	17.5	6.21	4.29	4.75

* Mass loss rate: $\left(\frac{m_{s0}\;-\;m_{s}}{m_{s0}}\right)\;\times \;100\%$, where *m*_*s*0_ and *m_s_* are the amounts of non-treated and the treated microspheres, respectively.

**Table 2:** Mass loss rates of MF_6_ in the acid-treatment reaction at the different treatment temperatures.

Temperature (°C)	20	40	60	80	100
**Parameter (*n*)**	6.0	6.0	6.0	6.0	6.0
* mass loss rate (wt %)	3.90	4.08	4.29	4.97	5.10

* Mass loss rate: $\left(\frac{m_{s0}\;-\;m_{s}}{m_{s0}}\right)\;\times \;100\%$, where *m*_*s*0_ and *m_s_* are the amounts of non-treated and the treated microspheres, respectively.

## Data Availability

The data presented in this study are available in this article and in the Supplementary Material.

## Supplementary Materials

The following are available online at https://www.mdpi.com/article/10.3390/nano11051337/s1, Figure S1: Impact of the acid-treatment on Raman spectra of MF*_n_* microspheres, Figure S2: Two extreme structures in MF*_n_* polymers, Figure S3: Two extreme structures in linear MF*_n_* polymers, Figure S4: Two ideal units in linear MF*_n_* polymer structure with the F/M molar ratio *n* = 1.5, Figure S5: Influence of polymerization degree *m* on the F/M molar ratio *n* in an ideal linear polymer structure, Figure S6: Influence of polymerization degree *m* on the F/M molar ratio *n* in the planar or three-dimensional structures, Figure S7: Linear structure of an MF*_n_* polymer molecule (Structure L), Figure S8: Planar structure of an MF*_n_* nonamer (Structure P_9_), Figure S9: Three-dimensional structure of an MF*_n_* seventy-two polymer (Structure T_72_), Figure S10: Terminal overreaction at the end of a linear MF*_n_* molecule (Equation LO), Figure S11: Terminal overreaction at the end of a planar MF*_n_* nonamer (Equation P_9_), Figure S12: Terminal overreaction at the end of a three-dimensional MF*_n_* seventy-two polymers (Equation T_72_), Figure S13: Silver concentration as a function of absorbance from spectrophotometry, Figure S14: SERRS performance of AgNPs treated with the liquor from MF_6_ acid-treatment reaction, Figure S15: SERRS spectra of a set of analytes collected on AgNP/MF_6_ substrate, Figure S16: SERRS spectrum as a function of the tetramethylthiuram disulfide concentration on the optimized AgNP/MF_6_, Figure S17: SERRS spectrum as a function of the malachite green concentration on the optimized AgNP/MF_6_, Figure S18: SERRS spectrum as a function of the p-hydroxythiophenol concentration on the optimized AgNP/MF_6_, Figure S19: SERRS spectrum as a function of the basic violet 14 concentration on the optimized AgNP/MF_6_, Table S1: Calculation of silver content in the AgNP/MF_3_.
