# Supplementary material for: SERRS Detection on Silver Nanoparticles Supported on Acid-Treated Melamine-Resin Microspheres
Source: Nanomaterials (Basel). 2021 May 19;11(5):1337. doi: 10.3390/nano11051337 (PMC8160733; doi:10.3390/nano11051337)
Supplement: Supplementary file 1 [file nanomaterials-11-01337-s001.zip › nanomaterials-1183351-supplementary.pdf]

nanomaterials

Article

SERRS Detection on Silver Nanoparticles Supported on Acid-Treated Melamine-Resin Microspheres

Chaofeng Duan [1], Lu Shen [1], Yuqing Guo [1], Xiaogang Wang [1], Xiaohua Wang [2] and Zhixian Hao [1,*]

1 Shanghai Key Laboratory of Chemical Assessment and Sustainability, School of Chemical Science and Engineering, Tongji University, Shanghai 200092, China; 1831027@tongji.edu.cn (C.D.); Shenlu218@163.com (L.S.); superyq@tongji.edu.cn (Y.G.); xgwang@tongji.edu.cn (X.W.)
2 Institute of Industrial Catalysis, Zhejiang University of Technology, Hangzhou 310014, China; wangxiaohua0909@163.com
* Correspondence: haozhixian@tongji.edu.cn; Tel.: +86-13761772106

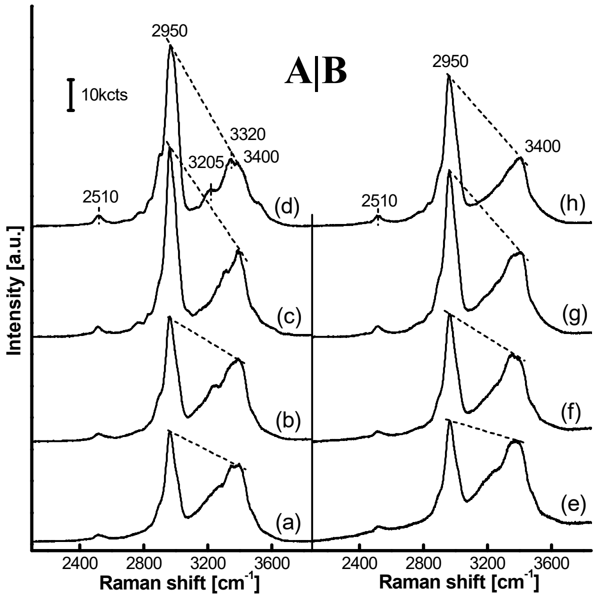

Figure S1. Impact of the acid-treatment on Raman spectra of MF_n microspheres. (**a**): MF_2, (**b**): MF_3, (**c**): MF_6 and (**d**): MF_9 in (**A**) were collected on non-treated microspheres, while (**e**): MF_2, (**f**): MF_3, (**g**): MF_6 and (**h**): MF_9 in (**B**) were collected on the acid-treated microspheres. An increasing parameter (n) enhanced the Raman scattering peak at 2950 cm^{-1} while the slope value between two scattering peaks (2950 and 3400 cm^{-1}) of each MF_n sample declined due to the acid-treatment reaction.

Figure S1A and B respectively show the Raman spectra of MF_n microspheres and the microspheres after the acid-treatment reaction. A strong Raman scattering band at 2950 cm^{-1} and a medium wide one at around 3400 cm^{-1} were due to the stretching vibrations of C–H and N–H that were attributed to the formaldehyde and melamine that had been added to the MF_n synthetic-reaction, respectively. The intensity ratio between the two bands, shown as a slope for each sample, was enhanced when the parameter (n) increased from 2.00 to 9.00 as shown from Figure S1A (a) to (d). This finding implies that there was a progressive addition of formaldehyde into the MF_n microspheres as the parameter (n) increased. Furthermore, the intensity ratio for each sample declined after the acid-treatment reaction, as shown by the peak slope from each sample in Figure 2A being higher than that from the sample with the same parameter (n) in Figure S1B. This suggests that the removed mass portion, more than 4.29 wt% as shown in Table 1, consisted of MF_n oligomers with more terminal $–NHCH_2OH$ groups. In addition, the Raman scattering band pattern at around 3400 cm^{-1} on each non-treated MF_n sample was complicated when

Citation: Duan, C.; Shen, L.; Guo, Y.; Wang, X.; Wang, X.; Hao, Z. SERRS Detection on Silver Nanoparticles Supported on Acid-Treated Melamine Resin-Microspheres. *Nanomaterials* **2021**, *11*, 1337. https://doi.org/10.3390/nano11051337

Received: 30 March 2021
Accepted: 10 May 2021
Published: 19 May 2021

Publisher's Note: MDPI stays neutral with regard to jurisdictional claims in published maps and institutional affiliations.

Copyright: © 2021 by the authors. Submitted for possible open access publication under the terms and conditions of the Creative Commons Attribution (CC BY) license (http://creativecommons.org/licenses/by/4.0/).

compared to that on the acid-treated sample. Scattering peaks at 3205 and 3320 cm^{-1} appeared faint after the acid-treatment reaction. This implied that oligomers had been removed during the acid-treatment reaction.

1. F/M Molar Ratio Present in MF$_n$ Polymer Structure

According to the valence bond theory, one molecule of formaldehyde combines two molecules of melamine at most in the molecular structure and one molecule of melamine needs six molecules of formaldehyde, as shown in Figure S2. Any possible F/M molar ratio in MF$_n$ polymer structures could thus be schemed out between the values 0.50 and 3.0 existent in Structures F$_{max}$ and M$_{max}$, respectively.

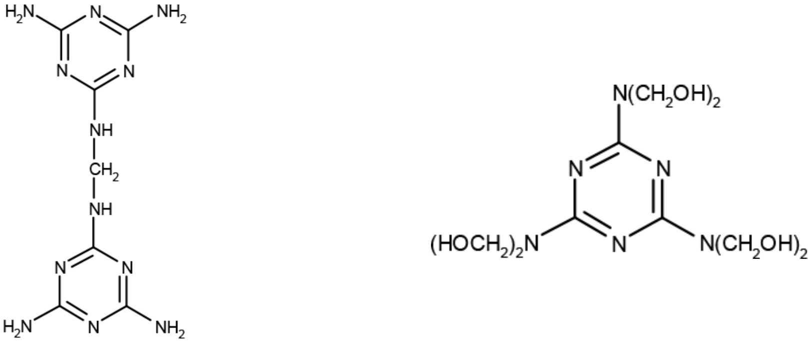

Structure F$_{max}$ (F/M molar ratio n = 0.50)   Structure M$_{max}$ (F/M molar ratio n = 3.0)

Figure S2. Two extreme structures in MF$_n$ polymers.

For example, various linear MF$_n$ polymers with the F/M molar ratio between 1.0 and 2.0 in Structures L$_1$ and L$_2$ in Figure S3 could be constructed, based on the –NH$_2$ side substitution with –NHCH$_2$OH groups in different degrees.

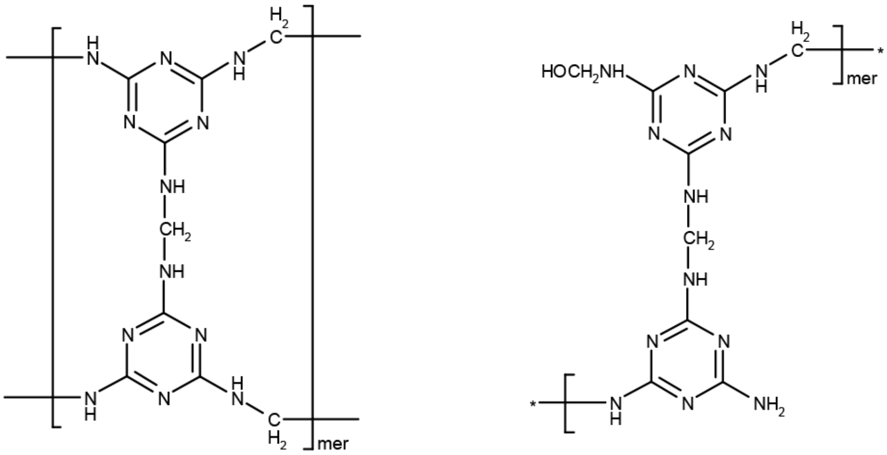

Structure L$_1$ (F/M molar ratio n = 1.0)   Structure L$_2$ (F/M molar ratio n = 2.0)

Figure S3. Two extreme structures in linear MF$_n$ polymers. Wherein, all the –NH$_2$ groups in the melamine structure are substituted by –NHCH$_2$–.

2. F/M Molar Ratio *n* Adopted in the Synthetic-Reaction, as well as Excess Monomer

The F/M molar ratio, i.e. the experiment parameter (n), in the synthetic-reaction for MF$_n$ polymers is another concept, which is hardly consistent with the F/M molar ratio (n) present in the resultant polymer structure. It is possible to synthesize MF$_n$ microspheres at the parameter n = 9.0, a value that is more than the maximum F/M molar ratio n = 6.0 in the Structure M$_{max}$ as shown in Figure S2. Even so, this excess formaldehyde in the synthetic-reaction was found to be crucial rather than useless. A concept "excess monomer" [26] was thus claimed to emphasize the impact of the parameter n on characteristics of the

resultant polymers. The excess monomer was verified to be able to influence various characteristics of the UF resin microspheres, e.g. output, solubility, crystallinity, nucleation, density and water absorption of UF resin microspheres, as reported in our previous work [26].

3. The Boundary Characteristics and Terminal Overreaction of Excess Monomer

According to the parameter (n), characteristics of the MF_n polymers are classifiable. The SERRS signals will gain intensity if the synthetic-reaction for the MF_n microspheres incorporated in the substrate is kept for 9.0 h with the parameter n more than 1.5, and otherwise it will be frustrate [27]. A similar phenomenon also occurred on the SERRS substrate incorporating UF microspheres, where the F/U molar ratio was more than 1.0 [26]. Obviously, the parameter $n = 1.5$ indeed acts as a boundary between two different SERRS performances of the substrate, on which the excess monomers are able to be divided into two regions for identification of the substrate performance. The parameter $n = 1.5$ is associated with two ideal units in MF_n structures as shown as Structure L_{mer} and T_{mer} in Figure S4, in which all $–NH_2$ are substituted by $–NHCH_2–$ groups or one $–NH_2$ group remained.

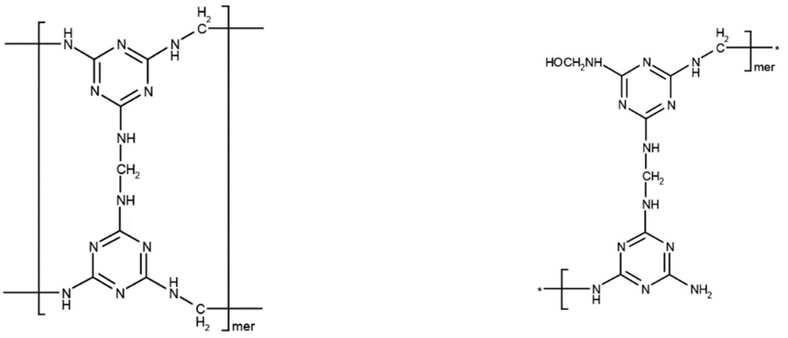

Structure T_{mer} $(2(C_3H_3N_6)\cdot 3(CH_2))$ Structure L_{mer} $(2(C_3H_4N_6)\cdot 2(CH_2)\cdot (CH_2O))$

Figure S4. Two ideal units in linear MF_n polymer structure with the F/M molar ratio $n = 1.5$.

The ideal unit Structure T_{mer} could extend in one, two or three-dimensions based on consistent $–NHCH_2–$ groups, except for the terminal sites as shown as Structures L, P_9 and T_{72} in Figure S7, S8 and S9. Formulas of MF_n polymers from it in one, two and three-dimensions are as following.

$$\text{Formula L: } [2(C_3H_3N_6)3(CH_2)]_x\ 2H_2O \qquad \text{(linear)} \tag{1}$$

$$\text{Formula P: } [2(C_3H_3N_6)3(CH_2)]_{xy}\ (2x + y - 1)(H_2O) \qquad \text{(planar)} \tag{2}$$

$$\text{Formula T: } [2(C_3H_3N_6)3(CH_2)]_{xyz}\cdot z(2x + y - 1)(H_2O) \qquad \text{(3D)} \tag{3}$$

Herein, the three-dimensional structure of Formula T is from overlay of the planar structure of Formula P in a lamellar way. The parameters x, y, z are also the numbers of replications in x-, y- and z-axis directions and the polymerization degree m equals x, xy, and xyz in linear, planar and three-dimensional structures, respectively.

The terminal overreaction of excess monomer emphasize a fact that the linear molecule $AB(AB)_mA$ from excess monomer A in the synthetic-reaction, for example, is different with the molecule $B(AB)_mAB$ from excess monomer B. For the linear, planar, and three-dimensional MF_n polymers, the terminal overreactions with excess formaldehyde are shown as following Equations.

Equation LO (linear)

$$[2(C_3H_3N_6)3(CH_2)]_x\cdot 2H_2O + 2CH_2O \Rightarrow [2(C_3H_3N_6)3(CH_2)]_x\ 2(H_2O\cdot CH_2O) \tag{4}$$

Equation PO (planar)

$$[2(C_3H_3N_6)3(CH_2)]_{xy}\cdot(2x+y-1)(H_2O)+(2x+y-1)(CH_2O) \Rightarrow [2(C_3H_3N_6)3(CH_2)]_{xy}\cdot(2x+y-1)(H_2O\cdot CH_2O) \quad (5)$$

Equation TO (3D)

$$[2(C_3H_3N_6)3(CH_2)]_{xyz}\cdot z(2x+y-1)(H_2O)+z(2x+y-1)CH_2O \Rightarrow [2(C_3H_3N_6)3(CH_2)]_{xyz}\cdot z(2x+y-1)(H_2O\cdot CH_2O) \quad (6)$$

After the terminal overreaction, Formula L, P and T are transferred into Formula LO, PO and TO as following.

$$\text{Formula LO: } [2(C_3H_3N_6)3(CH_2)]_{x}\cdot\times 2(H_2O\cdot CH_2O) \quad \text{(linear)} \quad (7)$$

$$\text{Formula PO: } [2(C_3H_3N_6)3(CH_2)]_{xy}\cdot(2x+y-1)(H_2O\cdot CH_2O) \quad \text{(planar)} \quad (8)$$

$$\text{Formula TO: } [2(C_3H_3N_6)3(CH_2)]_{xyz}\cdot\times z(2x+y-1)(H_2O\cdot CH_2O) \quad \text{(3D)} \quad (9)$$

These equations demonstrate that the excess monomer, e.g. excess formaldehyde, will further concentrate at the end of a linear polymer molecule, around a planar molecule or on the surfaces of a three-dimensional one under ideal structural conditions. Further demonstration of these overreactions is presented at the end of context, as Equations L_m, P_9 and T_{72} as shown in Figure S10, S11 and S12, respectively.

4. Influence of Polymerization Degree *m* on the F/M Molar Ratio *n* Present in MF_n Polymers

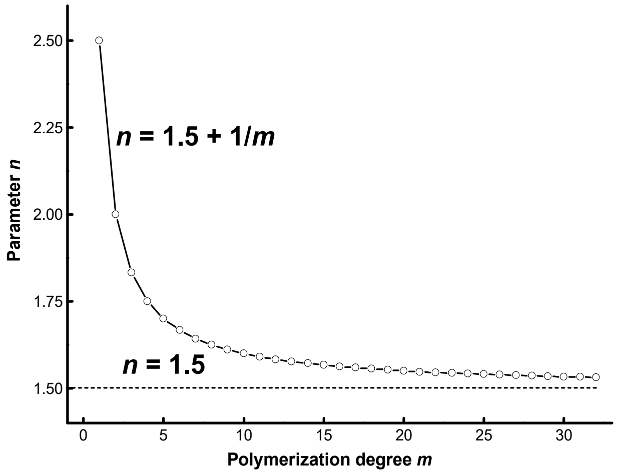

Figure S5. Influence of polymerization degree *m* on the F/M molar ratio n in an ideal linear polymer structure.

$$\text{Formula LO: } [2(C_3H_3N_6)3(CH_2)]_{x}\cdot 2(H_2O\cdot CH_2O);$$

$$\text{The F/M molar ratio } n = 1.5 + (1/x) = 1.5 + (1/m) \quad (10)$$

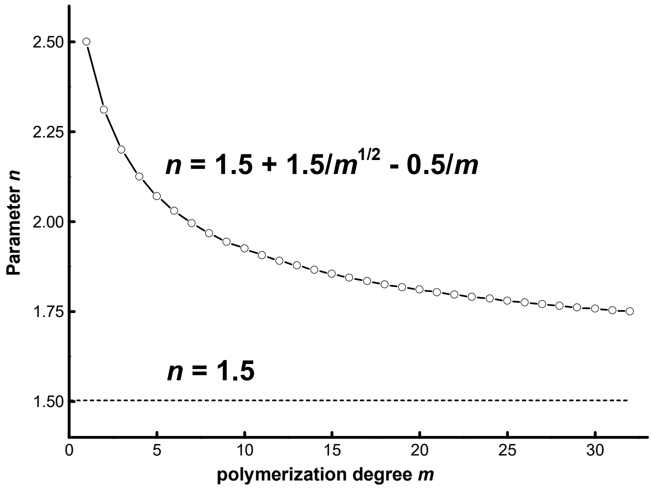

Figure S6. Influence of polymerization degree m on the F/M molar ratio *n* in the planar or three-dimensional structures.

$$\text{Formula PO: } [2(C_3H_3N_6)3(CH_2)]_{xx}\cdot(3x-1)(H_2O\cdot CH_2O); \quad (11)$$

$$\text{Formula TO: } [2(C_3H_3N_6)3(CH_2)]_{xxx}\cdot x(3x-1)(H_2O\cdot CH_2O) \quad (12)$$

Provided the $x = y = z$, the F/M molar ratio $n = 1.5 + 1.5/ - 0.5/x^2 = 1.5 + 1.5/m^{1/2} - 0.5/m$. The same function for Formulas PO and TO because Structure TO is from the lamellar overlay of Structure PO.

Influences of polymerization degree m on the F/M molar ratio n in one-, two- and three-dimensional structures are shown in Figure S5 and S6. Obviously, the F/M molar ratio n in MF_n polymers increases with the polymerization degree m decreasing under an excess formaldehyde condition, especially rapidly in the two- and three-dimensional cases.

5. Three Ideal Types of MF_n Polymer Structures and Their Terminal Overreactions

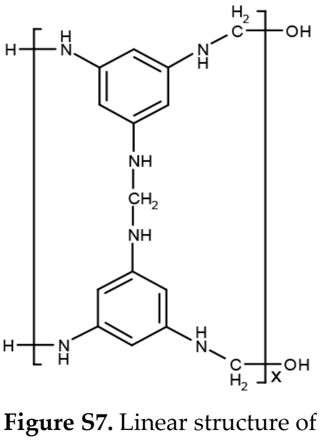

Figure S7. Linear structure of an MF_n polymer molecule (Structure L). x is the degree of polymerization.

$$\text{Formula L: } [2(C_3H_3N_6)3(CH_2)]_x \times 2H_2O; \quad \text{polymerization degree: } m = x \quad (13)$$

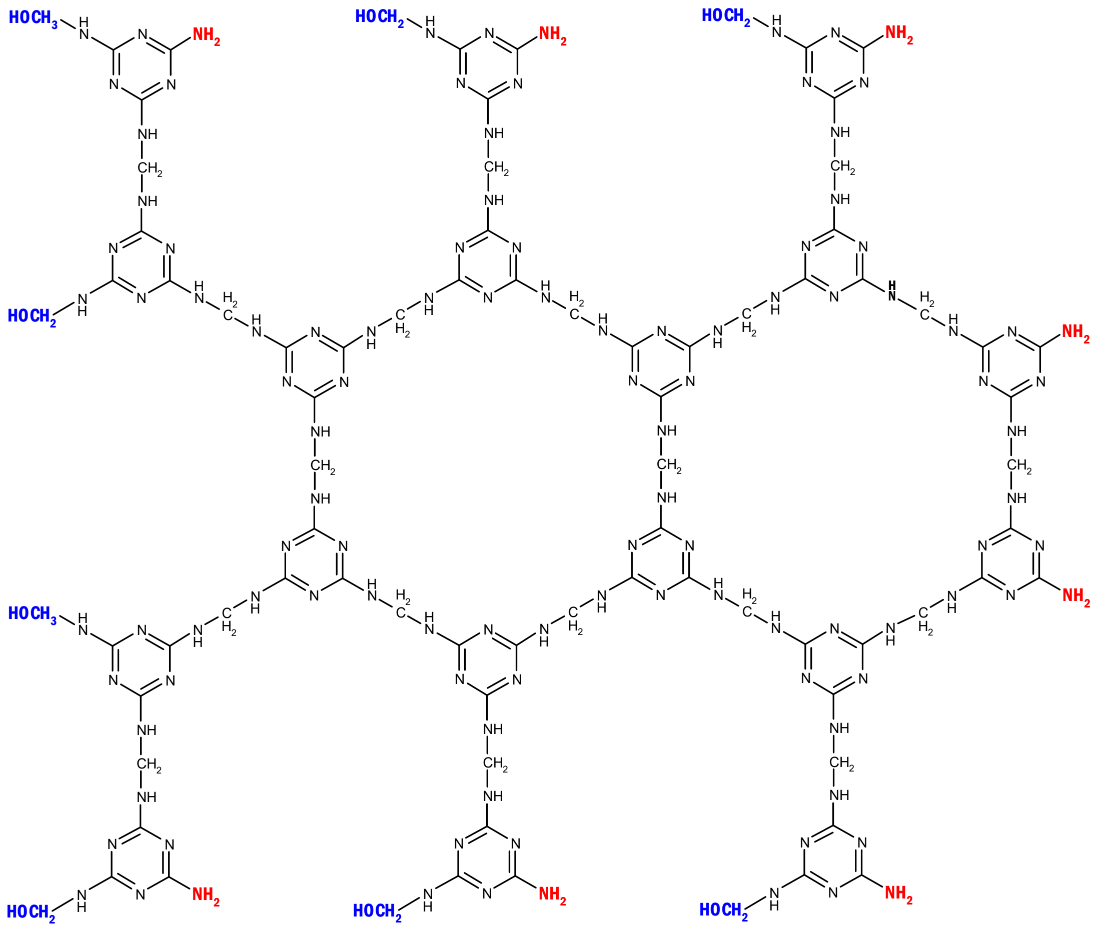

Figure S8. Planar structure of an MF_n nonamer (Structure P_9).

$$\text{Formula } P_9\text{: } [2(C_3H_3N_6)3(CH_2)]_9\ 8(H_2O);\ x = y = 3;\quad \text{polymerization degree: } m = xy = 9 \tag{14}$$

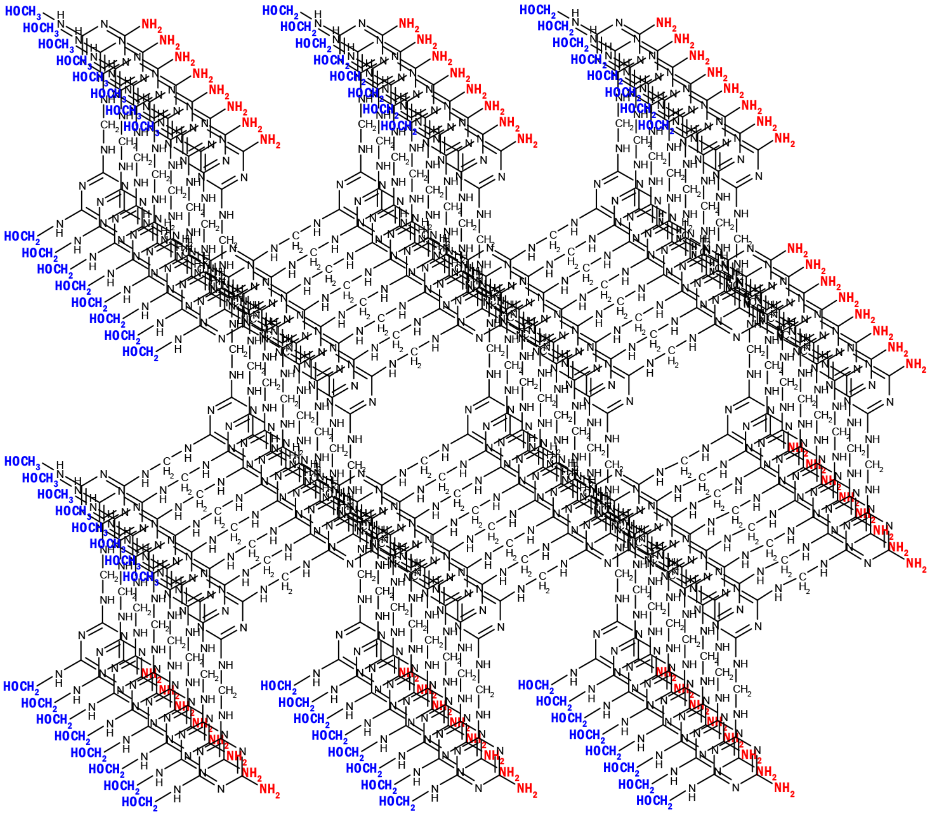

Figure S9. Three-dimensional structure of an MF_n seventy-two polymer (Structure T_{72}).

$$\text{Formula } T_{72}\text{: } [2(C_3H_3N_6)3(CH_2)]_{72}\ 64(H_2O);\ x = y = 3;\ z = 8; \\ \text{polymerization degree } m = xyz = 72 \quad (15)$$

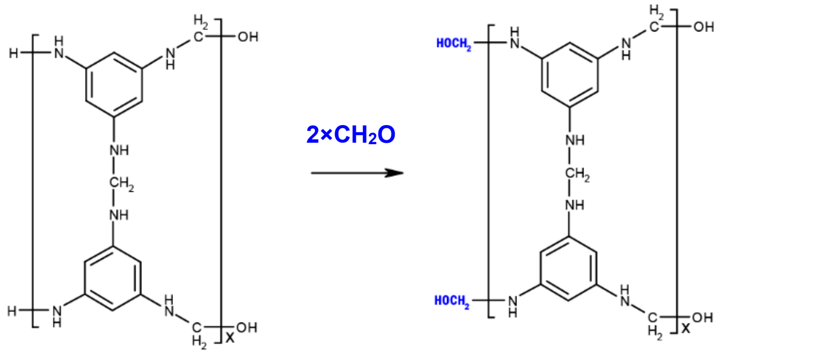

$[2(C_3H_3N_6)\cdot3(CH_2)]_x\cdot2H_2O$ $[2(C_3H_3N_6)\cdot3(CH_2)]_x\cdot2(CH_2O\cdot H_2O)$

Figure S10. Terminal overreaction at the end of a linear MF_n molecule (Equation LO); polymerization degree $m = x$.

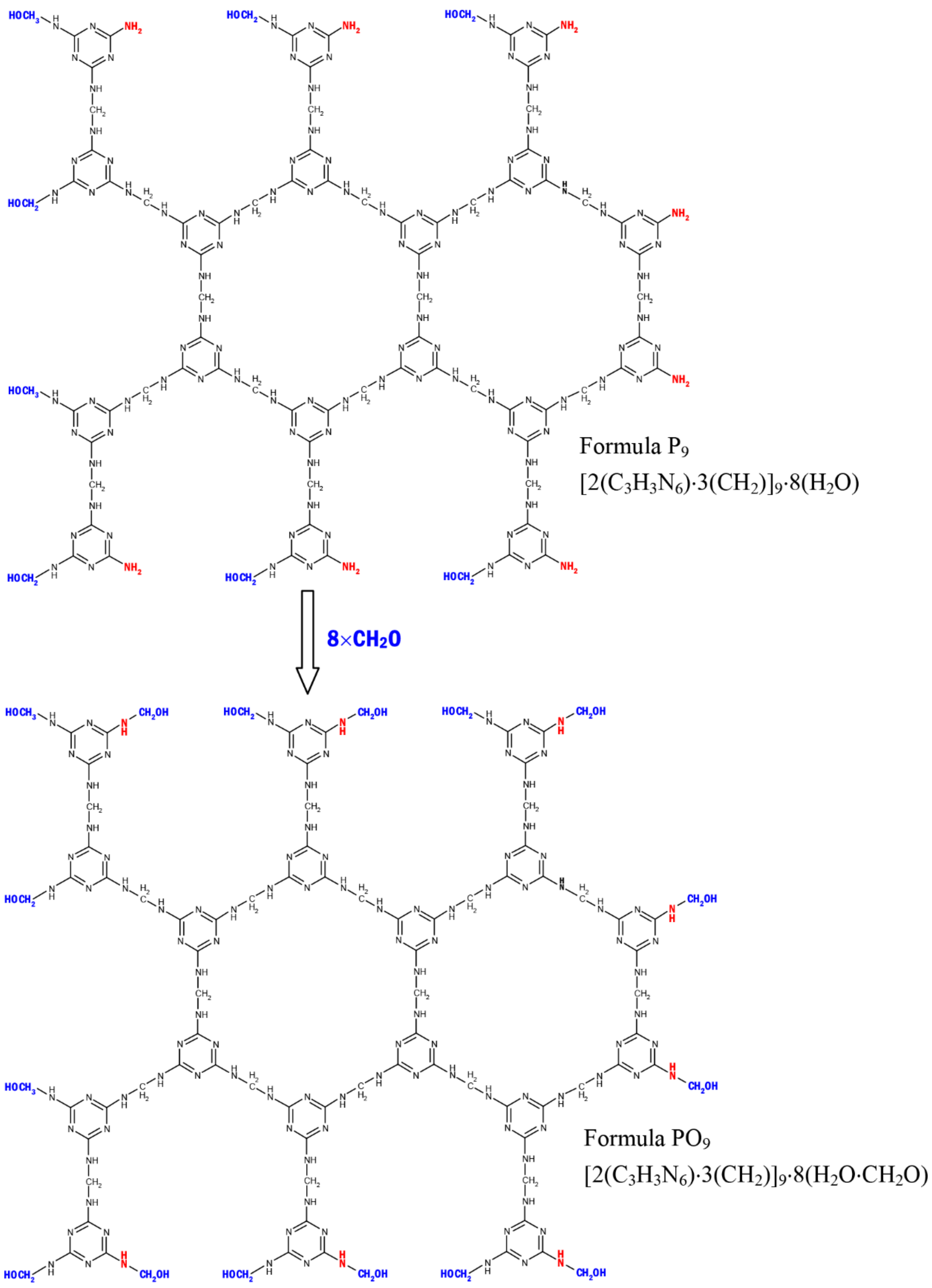

Figure S11. Terminal overreaction at the end of a planar MF_n nonamer (Equation P_9); $x = y = 3$; polymerization degree $m = xy = 9$.

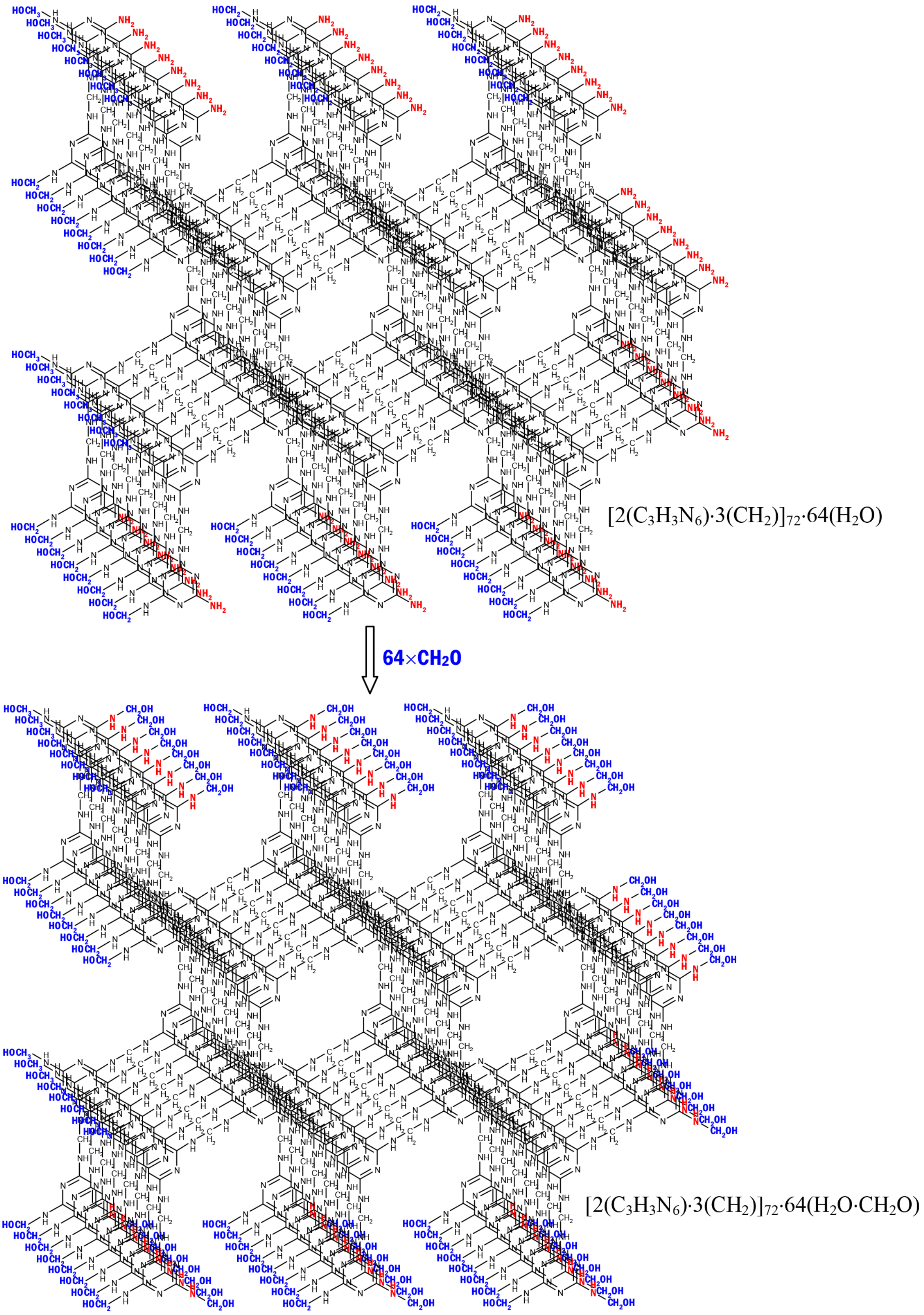

Figure S12. Terminal overreaction at the end of a three-dimensional MF$_n$ seventy-two polymers (Equation T$_{72}$) $x = y = 3$, $z = 8$; polymerization degree $m = xyz = 72$.

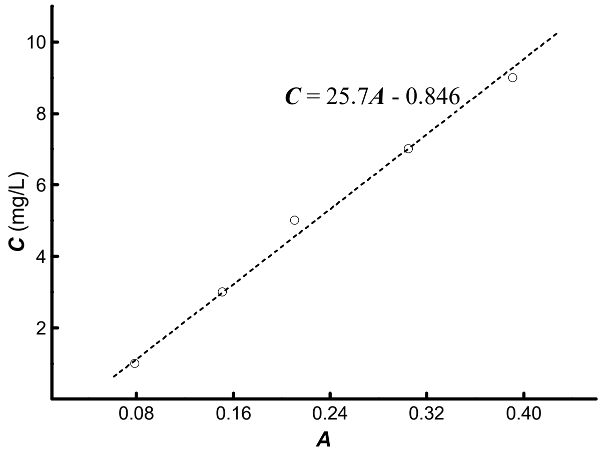

Figure S13. Silver concentration as a function of absorbance from spectrophotometry.

The working curve: $C = 25.7A - 0.846$ (mg/L), for which the wavelength 400 nm was adopted

In order to acquire the AgNP content in AgNP/MF_3, 100 mg MF_3 was added in 10.0 mL AgNP colloid solution with a silver concentration 5.00 mg/L and let settlement for 1 h following a 5 min ultrasound treatment. The mixture was then separated at a centrifugal rate of 6000 rpm for 5 min. AgNP concentrations in the initial AgNP colloid solution and the residual colloid liquor after the AgNP adsorption were both measured on absorption spectrophotometry, respectively. The silver content in the AgNP/MF_3 was calculated and summarized in Table S1.

Table S1. Calculation of silver content in the AgNP/MF_3.*

	**incorporating no-treated MF_3**			**incorporating acid-treated MF_3**		
	A	*C*(mg/L)	*m*(mg)	*A*	*C* (mg/L)	*m*(mg)
colloid before adsorption	0.227	5.00	0.0500	0.227	5.00	0.0500
colloid after adsorption	0.192	4.09	0.0409	0.205	4.42	0.0442
Ag adsorbed from 10.0 mL colloid	0.0500 − 0.0409 = 0.0091 (mg)			0.0500 − 0.0442 = 0.0058 (mg)		
Ag content in 100 mg AgNP/MF_3	0.0091 (mg)/100 (mg)= 91×10^{-6}			0.0068 (mg)/100 (mg)=58×10^{-6}		

* The silver concentration of the colloid solution: $C = 25.7A - 0.846$ (mg/L); The silver amount in the 10.0 mL colloid solution: $m = C \times 10.0 \times 10^{-3}$(mg).

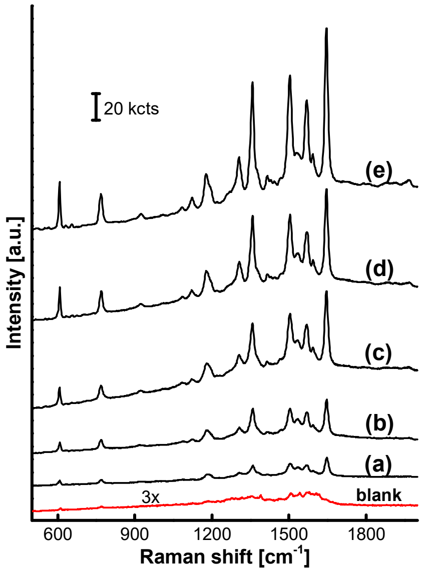

Figure S14. SERRS performance of AgNPs treated with the liquor from MF6 acid-treatment reaction. (**a**) 20, (**b**) 40, (**c**) 60, (**d**) 80 and (**e**) 100 °C were collected on the AgNPs that were treated with liquors from the MF6 acid-treatment reaction at varied temperatures from 20 to 100 °C and a laser power of 0.01 mW. The blank AgNPs was examined at a laser power of 0.05 mW and its R6G-SERRS signal was magnified 3 times as shown at the bottom. SERRS signals on the AgNPs had their intensities gradually enhanced with the MF6 acid-treatment temperature increased.

Figure S14 shows the R6G-SERRS performance of AgNPs treated with the liquor from the MF_6 acid-treatment reaction. The R6G SERRS signal was enhanced with the acid-treatment temperature gradually increased from 0 to 100°C. These results were on the contrary with those obtained on the AgNP/MF_6 where the incorporating MF_6 from the same acid-treatment reactions executed at the varied temperature from 40 to 100°C. It was obvious that the oligomers in the MF_n microspheres had transferred into the hydrolyzed liquor and played an important role in the construction of SERRS hotspots.

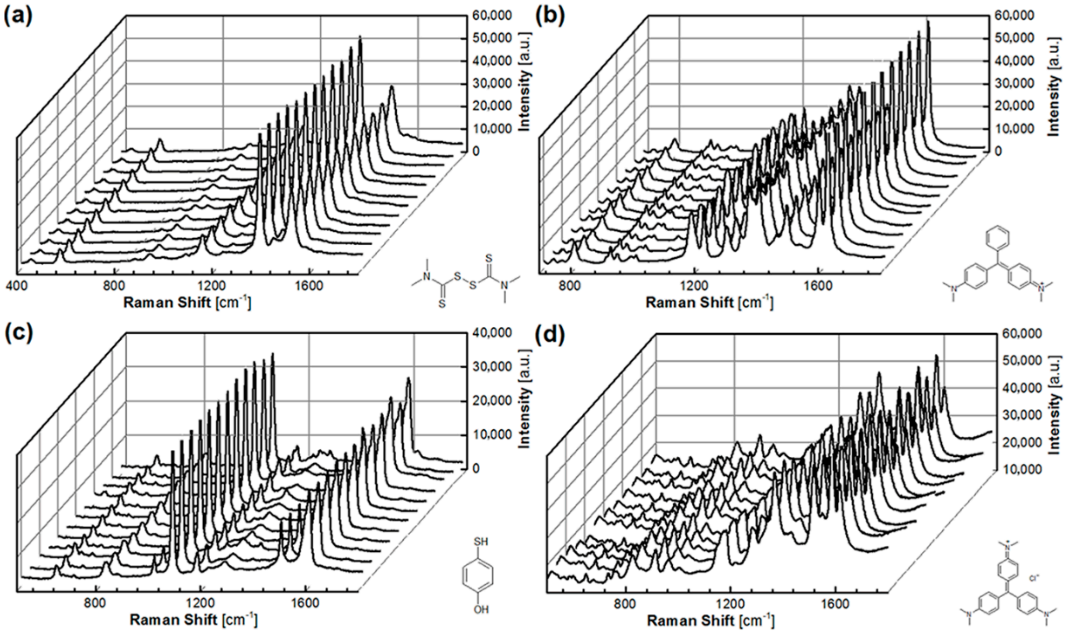

Figure S15. SERRS spectra of a set of analytes collected on AgNP/MF_6 substrate. (**a**) Tetramethylthiuram disulfide, (**b**) malachite green, (**c**) p-hydroxythiophenol and (**d**) basic violet 14 were collected on substrate incorporating MF_6 microspheres, synthesized at pH 4.0 and 65 °C for 20 min, acid-treated at pH 4.0 and 40 °C for 10 min, where twelve spectra for statistic analysis of each analyte were randomly collected on different microspheres of the SERRS substrate.

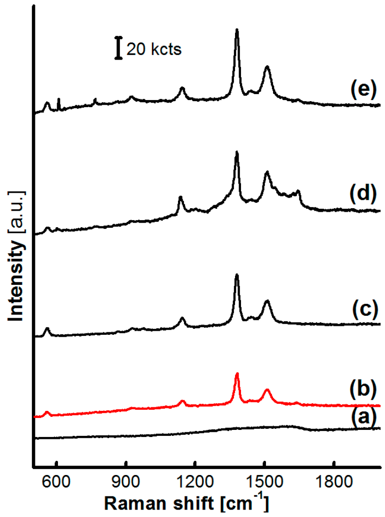

Figure S16. SERRS spectrum as a function of the tetramethylthiuram disulfide concentration on the optimized AgNP/MF6. (**a**) 1.0 × 10^{-9}, (**b**) 1.0 × 10^{-8}, (**c**) 1.0 × 10^{-7}, (**d**) 1.0 × 10^{-6} and (**e**) 1.0 × 10^{-5} M were tetramethylthiuram disulfide -SERRS spectra on the optimized AgNP/MF6 treated with a tetramethylthiuram disulfide solution from 1.0 × 10^{-9} to 1.0 × 10^{-5} M tetramethylthiuram disulfide at a laser power of 0.01 mW. The tetramethylthiuram disulfide SERRS spectrum was still clear if the AgNP/MF6 was treated with a 1.0 × 10^{-8} M tetramethylthiuram disulfide solution.

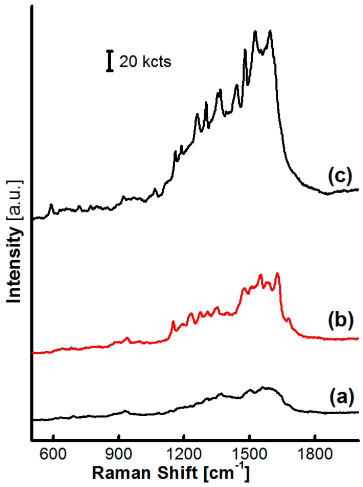

Figure S17. SERRS spectrum as a function of the malachite green concentration on the optimized AgNP/MF6. (**a**) 1.0 × 10^{-8}, (**b**) 1.0 × 10^{-7} and (**c**) 1.0 × 10^{-6} M were malachite green-SERRS spectra on the optimized AgNP/MF6 treated with a malachite green solution from 1.0 × 10^{-8} to 1.0 × 10^{-6} M malachite green at a laser power of 0.01 mW. The malachite green SERRS spectrum was clearest if the AgNP/MF6 was treated with a 1.0 × 10^{-6} M concentration malachite green solution.

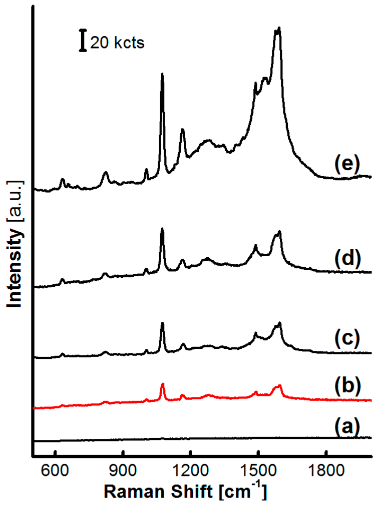

Figure S18. SERRS spectrum as a function of the p-hydroxythiophenol concentration on the optimized AgNP/MF_6. (**a**) 1.0 × 10^{-8}, (**b**) 1.0 × 10^{-7}, (**c**) 1.0 × 10^{-6}, (**d**) 1.0 × 10^{-5} and (**e**) 1.0 × 10^{-4} M were p-hydroxythiophenol-SERRS spectra on the optimized AgNP/MF_6 treated with a p-hydroxythiophenol solution from 1.0 × 10^{-8} to 1.0 × 10^{-4} M p-hydroxythiophenol at a laser power of 0.01 mW. The p-hydroxythiophenol SERRS spectrum was still clear if the AgNP/MF_6 was treated with a 1.0 × 10^{-7} M p-hydroxythiophenol solution.

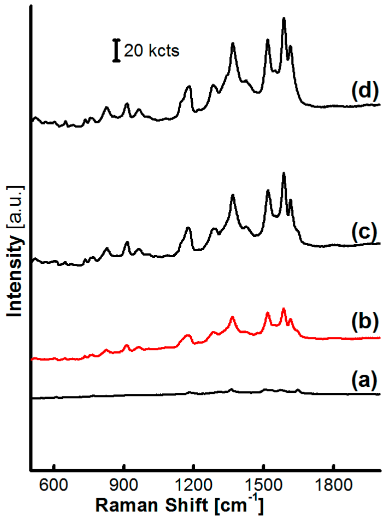

Figure S19. SERRS spectrum as a function of the basic violet 14 concentration on the optimized AgNP/MF_6. (**a**) 1.0 × 10^{-11}, (**b**) 1.0 × 10^{-9}, (**c**) 1.0 × 10^{-7} and (**d**) 1.0 × 10^{-5} M were basic violet 14-SERRS spectra on the optimized AgNP/MF_6 treated with a basic violet 14 solution from 1.0 × 10^{-11} to 1.0 × 10^{-5} M basic violet 14 at a laser power of 0.01 mW. The basic violet 14 SERRS spectrum was still clear if the AgNP/MF_6 was treated with a 1.0 × 10^{-9} M basic violet 14 solution.
